# ERM Proteins Play Distinct Roles in Cell Invasion by Extracellular Amastigotes of *Trypanosoma cruzi*

**DOI:** 10.3389/fmicb.2017.02230

**Published:** 2017-11-21

**Authors:** Éden R. Ferreira, Alexis Bonfim-Melo, Esteban M. Cordero, Renato A. Mortara

**Affiliations:** ^1^Departamento de Microbiologia, Imunologia e Parasitologia, Escola Paulista de Medicina, Universidade Federal de São Paulo, São Paulo, Brazil; ^2^Centro de Genómica y Bioinformática, Facultad de Ciencias, Universidad Mayor, Santiago, Chile

**Keywords:** *Trypanosoma cruzi*, extracellular amastigote, ERM proteins, Host cell invasion, actin cytoskeleton

## Abstract

The protozoan parasite *Trypanosoma cruzi* is the causative agent of Chagas' disease. In mammalian hosts, *T. cruzi* alternates between trypomastigote and amastigote forms. Additionally, trypomastigotes can differentiate into amastigotes in the extracellular environment generating infective extracellular amastigotes (EAs). Ezrin-radixin-moesin (ERM) are key proteins linking plasma membrane to actin filaments, the major host cell component responsible for EA internalization. Our results revealed that depletion of host ezrin and radixin but not moesin inhibited EAs invasion in HeLa cells. ERM are recruited and colocalize with F-actin at EA invasion sites as shown by confocal microscopy. Invasion assays performed with cells overexpressing ERM showed increased EAs invasion in ezrin and radixin but not moesin overexpressing cells. Finally, time-lapse experiments have shown altered actin dynamics leading to delayed EA internalization in ezrin and radixin depleted cells when compared to control or moesin depleted cells. Altogether, these findings show distinct roles of ERM during EAs invasion, possibly regulating F-actin dynamics and plasma membrane interplay.

## Introduction

The parasitic protozoan *Trypanosoma cruzi* is the causative agent of Chagas' disease that affects 6–7 million people worldwide, mostly in the South and Central America, although the incidence has increased in other continents due to migration of infected people (WHO, 2017). Additionally, Chagas' disease was responsible for 76% of all deaths caused by Neglected Tropical Diseases in Brazil from 2000 to 2011 (Martins-Melo et al., 2016). In mammalian hosts, *T. cruzi* alternates between extracellular (infective) trypomastigote forms and intracellular (replicative) amastigote forms (Vianna, 1911; Dvorak and Hyde, 1973). Alternatively, trypomastigotes can also differentiate into amastigotes in the extracellular environment generating infective extracellular amastigotes (EAs) (Andrews et al., 1987; Ley et al., 1988; Mortara, 1991). Host cell invasion by these forms is mediated by complex cellular signaling events triggered by parasite surface proteins and secreted molecules (reviewed in Mortara et al., 2005; reviewed in Ferreira et al., 2012, 2016) leading to actin filament reorganization, the main event in EA uptake. During EA internalization, the participation of actin cytoskeleton regulators, such as Rac1, gelsolin (Procópio et al., 1999; Fernandes and Mortara, 2004) and other proteins related to actin cytoskeleton regulation have been demonstrated to colocalize with actin at parasite invasion sites (Procópio et al., 1999; Bonfim-Melo et al., 2015). Additionally, host cell membrane components participate in EA invasion (Fernandes et al., 2007, 2013).

Ezrin-radixin-moesin (ERM) proteins are key elements linking actin filaments to the plasma membrane (Bretscher et al., 2002), important for diverse cellular processes, such as maintenance of cell morphology and cell migration (Ivetic and Ridley, 2004). ERM proteins (ezrin, radixin, and moesin) display very similar domain structure with two conformational states and cellular locations: closed (inactive) forms are cytoplasmic whereas in opened (active) state they localize at the cell membrane (Turunen et al., 1994). When opened, ERM proteins expose the actin-binding site at the C-terminal region, and at the N-terminal region, the binding domain links the molecule to the plasma membrane. Binding of inner leaflet PIP_2_ as well as phosphorylation of a threonine at C-terminal region (T567, T564, and T558 in ezrin, radixin and moesin, respectively) disrupts intramolecular interaction between both domains leading to ERM proteins activation (Hao et al., 2009; Bosk et al., 2011). Several studies report the importance of ERM proteins in diverse organisms and cells. The lack of moesin in *Drosophila melanogaster* impairs epithelial cell-cell junction, ezrin knockout mice die few days after birth and radixin depleted mice present hyperbilirubinemia, develop cochlear stereocilia degeneration and consequent deafness (Kikuchi et al., 2002; Speck et al., 2003; Kitajiri et al., 2004; Saotome et al., 2004). ERM proteins are also described to participate in infection by diverse intracellular bacterial pathogens such *Helicobacter pylori, Neisseria meningitidis, Shigella flexneri*, enteropathogenic and enterohemorrhagic *Escherichia coli* (Skoudy et al., 1999; Goosney et al., 2001; Eugène et al., 2002; Selbach et al., 2004). Regarding protozoan pathogens little is known about the involvement of ERM proteins. Two reports described the participation of ERM proteins during infection of *Theileria annulata* in bovine macrophages (Baumgartner, 2011; Ma and Baumgartner, 2014). In the present study, we demonstrate that ERM proteins, ezrin and radixin, are involved in *T. cruzi* EAs invasion process.

## Materials and methods

### Parasites and mammalian cells

*Trypanosoma cruzi* G strain isolate (DTU I) (Yoshida, 1983; Zingales et al., 2009; Lima et al., 2013) was used in this study. EAs were obtained by differentiation of tissue culture trypomastigotes (TCTs) in LIT medium at pH 5.8 for 14 h as previously described (da Silva et al., 2006). HeLa cells (Instituto Adolfo Lutz, São Paulo, SP, Brazil) were grown in RPMI 1640 medium (Sigma-Aldrich) supplemented with 10% fetal bovine serum (FBS, Invitrogen), 10 μg mL^−1^ streptomycin, 100 U mL^−1^ penicillin and 40 μg mL^−1^ gentamicin at 37°C and 5% CO_2_.

### Lentiviral transduction and HeLa lineage establishment

Lentiviral vectors were produced as previously described in Bonfim-Melo et al. (2015) and target sequences were acquired from Sigma-Aldrich: Ezrin/NM_003379 (Cat. no. TRCN0000380178), sequence: GTA CCG GTG ATG CCC TTG GAC TGA ATA TCT CGA GAT ATT CAG TCC AAG GGC ATC ATT TTT TG, Radixin/NM_002906 (Cat. no. TRCN0000415784), sequence: CCG GAT GAG CAT GAC GAC AAG TTA ACT CGA GTT AAC TTG TCG TCA TGC TCA TTT TTT TG, Moesin/NM_002444 (Cat. no. TRCN0000062411), sequence: CCG GGC ATT GAC GAA TTT GAG TCT ACT CGA GTA GAC TCA AAT TCG TCA ATG CTT TTT G. For lentiviral packaging and production 5 × 10^6^ HEK293T cells were plated on 10 cm (diameter) plates containing DMEM with 10% FCS, 0.2 mM glutamine, 10 U mL^−1^ penicillin and 10 μg mL^−1^ streptomycin. One day after plating, cells were incubated with DMEM without serum and 15 μg of shRNAi (PLKO.1 + target sequence) vector, 10 μg of viral protein plasmid (pdR8.9) and 5 μg of VSV-G (pCI-VSVG) then transfected by calcium phosphate co-precipitation method. After 6 h, the cells were incubated glycerol 15% in PBS (shock) for 2 min, washed twice with PBS and incubated with DMEM 10% FCS. After 24 and 48 h the cell culture supernatant were harvested and stored at −80°C. For shRNAi transduction 5 × 10^4^ HeLa cells were plated in 6 well plates, incubated with 2 mL of virus rich HEK293T culture medium and 8 μg mL^−1^ of polybrene® (Sigma-Aldrich). For transduced cell selection, 48 h after transduction HeLa cells were incubated with increasing concentrations of puromycin (0.2–10 μg mL^−1^) within 2 weeks. To evaluate shRNAi transduction efficiency, cells were washed with PBS and lysis buffer added (50 mM Tris-HCl, 150 mM NaCl, 1 mM EDTA, 1% Triton X-100, protease inhibitor cocktail 1 ×, Thermo Scientific) and centrifuged at 16,000 × *g*, 5 min and 4°C. Supernatant was collected and quantified by the Bradford method (Bradford, 1976). Next, 30 μg of cell lysates were ran in 10% SDS-PAGE and protein expression was evaluated by Western blot using anti-ezrin 1:2,000 (Sigma-Aldrich, cat. no. E8897), anti-radixin 1:2,000 (Sigma-Aldrich, cat. no. R3653) or anti-moesin 1:1,000 (Santa Cruz cat. no. sc-6410) incubated overnight at 4°C. Anti-actin (Cell signaling cat. no. 4967) or anti-GAPDH (Cell signaling cat. no. 2118S) were used as loading controls at a 1:5,000 dilution. Secondary antibodies (Sigma Aldrich) were incubated 1 h at room temperature at a dilution of 1:10,000. All antibodies solutions and blocking steps were carried out in PBS-Tween 20 0.1% + 5% bovine serum albumin (Sigma-Aldrich). Bound antibody signals were amplified with ECL (GE Healthcare) and luminescent bands visualized in an Alliance 2.7 photo documenter (UVItec).

### Invasion and recruitment assays

For cells depleted for ERM proteins, assays were performed by adding 500 μL of cell suspension (1.5 × 10^5^) to 24 well plates containing sterile glass coverslips and incubated overnight at 37°C and 5% CO_2_. In the following day parasites (MOI 10:1) were added and the plates incubated for another 2 h at 37°C in a 5% CO_2_ humidified incubator. Cells were then gently washed 5 times with PBS to remove unattached parasites, fixed with Bouin and stained with Giemsa as previously described in Ferreira et al. (2016).

For invasion assays with cells overexpressing mutant ERM constructs, 2 × 10^5^ HeLa cells were plated in 13 mm sterile glass coverslips on 6 well plates. In the next day, 3 μg of plasmids were incubated with 6 μL of FuGENE HD (Promega) in 100 μL of Opti-MEM (Gibco) for 20 min and then added to the wells containing cells in 1 mL of serum free RPMI. After 9 h cells were washed and incubated with fresh RPMI + 10% FCS medium. After 48 h the coverslips were transferred to 24 well plates following 2 h AE incubation (MOI 10:1) at 37°C, 5% CO_2_. Next, coverslips were washed 5 times with PBS to remove unattached parasites, fixed with paraformaldehyde 4% in PBS, washed 2 times with PBS after 20 min and incubated with blocking and permeabilizing solution PGS (0.2% gelatin, 0.1% saponin and 0.1% NaN_3_, diluted in PBS), containing 1 μg mL^−1^ 4′,6-diamidino-2-phenylindole (DAPI, Sigma-Aldrich). After 1 h, coverslips were mounted in glycerol buffered with 0.1 M Tris, pH 8.6, with 0.1% *p*-phenylenediamine as anti-fade agent. For cells transfected with radixin containing plasmids anti-HA antibody (Santa Cruz) was diluted 1:100 and subsequently reacted with Alexa Fluor-488 anti-mouse IgG (Invitrogen) 1:200 as secondary antibody. Internalized parasites were quantified under epifluorescence microscopy (Olympus BX51). pEGFP-N1_EZRIN_WT, pEGFP-N1_EZRIN_T567A, pEGFP-N1_EZRIN_T567D (Ren et al., 2012) were kindly provided by Dr. Chand Khanna, National Cancer Institute, USA. pF-HA_RADIXIN_WT, pF-HA_RADIXIN_T567A, pF-HA_RADIXIN_T564E (Grimsley et al., 2006) were kindly provided by Dr. Kodi Ravichandran, University of Virginia, USA. pHJ320 (Moesin wt), pHJ321 (Moesin T558A), pHJ322 (Moesin T558D) (Hao et al., 2009) were purchased from Addgene.

To evaluate recruitment and colocalization with actin, HeLa cells transfected with ERM mutated constructs were incubated with EAs (MOI 10:1) for 1 h and stained with phalloidin-TRITC diluted 1:1,000 (Sigma-Aldrich) according to the immunofluorescence protocol described above. Images were acquired with a TCS SP5 II Tandem Scanner confocal microscope (Leica Microsystems, Wetzlar, Germany) using a 63 × NA 1.40 PlanApo oil immersion objective and processed with Imaris software 7.0 (Bitplane).

### Phosphoprotein assays

HeLa cells (5 × 10^6^) were seeded onto 10 cm (diameter) plates and grown for 24 h. Cells were incubated with serum-free RPMI for another 24 h (starvation) parasite were added for the following time periods: 0 (without contact), 5, 30, 60, and 90 min. Following incubation, cells were washed with PBS, harvested with a cell scraper in a solution of cold PBS containing 2 mM Na_3_VO_4_ and NaF), centrifuged and lysed with cell lysis buffer containing 5 mM of Na_3_VO_4_ and 2 mM NaF. Protein quantification was performed using Bradford assay (Bradford, 1976). Subsequently, samples were submitted to 10% SDS-PAGE, transferred to nitrocellulose membranes and processed as described above. Anti-pERM (1:1,000, Sigma-Aldrich, Cat no. SAB4504260) was incubated for overnight at 4°C, followed by 1 h secondary antibodies (1:10,000, Sigma-Aldrich) incubation. Bound antibodies were visualized as described above.

### Time-lapse analysis and confocal microscopy

For time lapse analysis of live cells, HeLa cells were plated in Hi-Q4 dishes (ibidi) and, 24 h later, transfected with LifeAct-RFP® (ibidi) and FuGENE HD (Roche), according to the manufacturer's instructions. 48 h post-transfection cells were stained with CMFDA (Invitrogen, 5 μM) for 30 min at 37°C. Time-lapse acquisition was performed under physiological conditions (humidified atmosphere at 37°C and 5% CO_2_) in a TCS SP5 II Tandem Scanner (Leica) confocal microscope with a 63 × NA 1.40 PlanApo oil immersion objective. Image processing, analysis and multidimensional reconstructions were performed with Imaris 7.0 software (Bitplane) and ImageJ (Schneider et al., 2012).

### Statistical analysis

Statistical analyses were performed with GraphPad Prism® employing Student's *t*-test. Data are presented as mean and each dot represents a replicate. ^*^P < 0.05, ^**^P < 0.005, and ^***^P < 0.001 mean significance.

## Results

### Depletion of ezrin, radixin but not moesin inhibits EA host cell invasion

**Figure 1:**
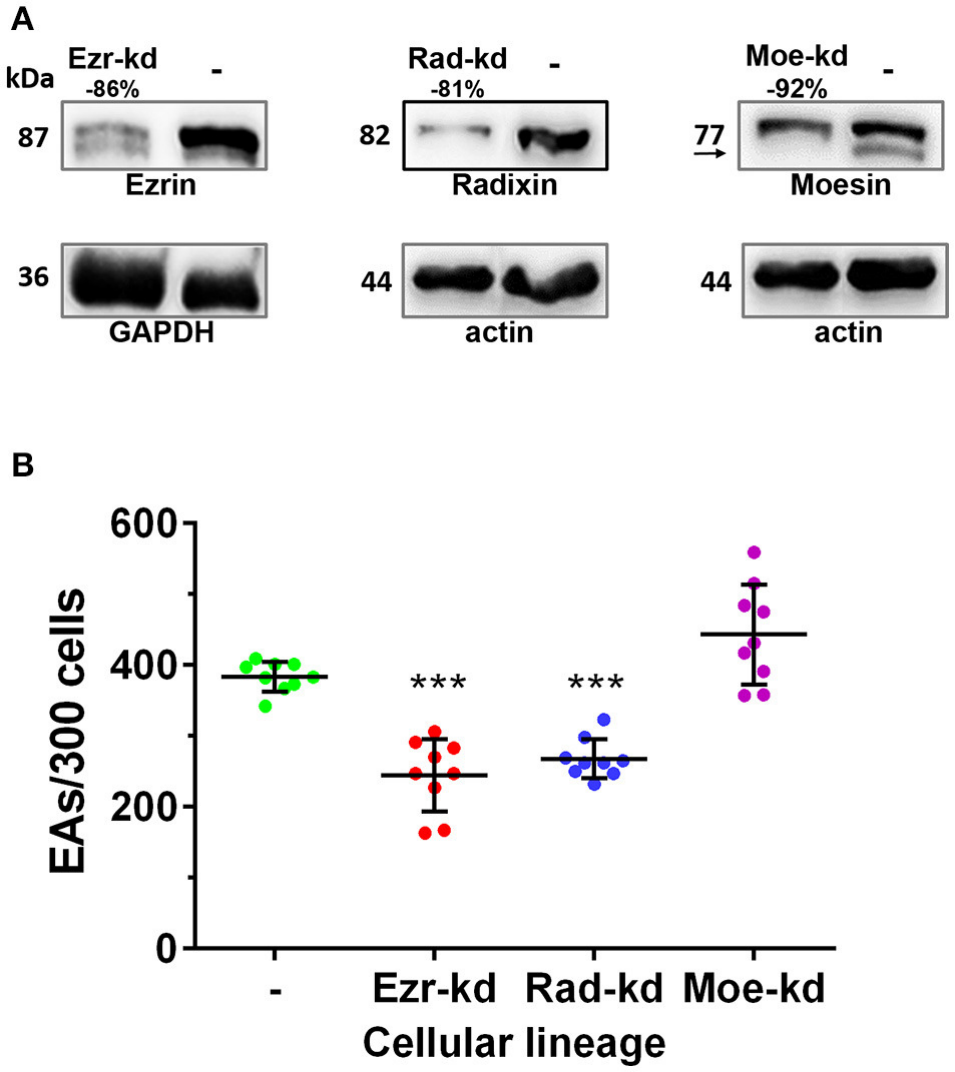
Depletion of ezrin and radixin but not moesin inhibits parasite invasion. **(A)** HeLa cells were submitted to lentiviral transduction with shRNAi sequences for ezrin, radixin or moesin. After selection and expansion of transduced cell cultures, the protein expression was evaluated by western blotting. Percentages represent the relative depletion of ezrin, radixin or moesin over the amount of Loading control (GAPDH or actin). **(B)** Cells were incubated with EAs (MOI 10:1) for 2 h followed by Bouin fixation and Giemsa staining. The experiment was performed in triplicate (three coverslips per group in each experiment) and intracellular parasites were counted in 300 cells/coverslip. Control groups (-) (non-transduced HeLa cells). This result is the mean and standard deviation of three independent experiments ±SD. ^***^P < 0.001.

ERM participation during pathogen invasion has been previously reported (Skoudy et al., 1999; Eugène et al., 2002; Selbach et al., 2004; Baumgartner, 2011), but so far their role in *T. cruzi* invasion has never been investigated. Since EAs depend on the actin cytoskeleton to invade host cells and ERM proteins participate in actin cytoskeleton regulation we investigated whether ERM proteins could modulate EA invasion. To answer this question, invasion assays using HeLa cells depleted to ezrin, radixin or moesin were performed. Our results demonstrated that depletion of ezrin and radixin inhibits EA invasion (Figure 1B). Different from ezrin and radixin, depletion of moesin did not affect parasite invasion rates. Depletion levels of ezrin radixin and moesin can be found in Figure 1A.

### Ezrin, radixin, and moesin are recruited to EAs invasion sites

**Figure 2:**
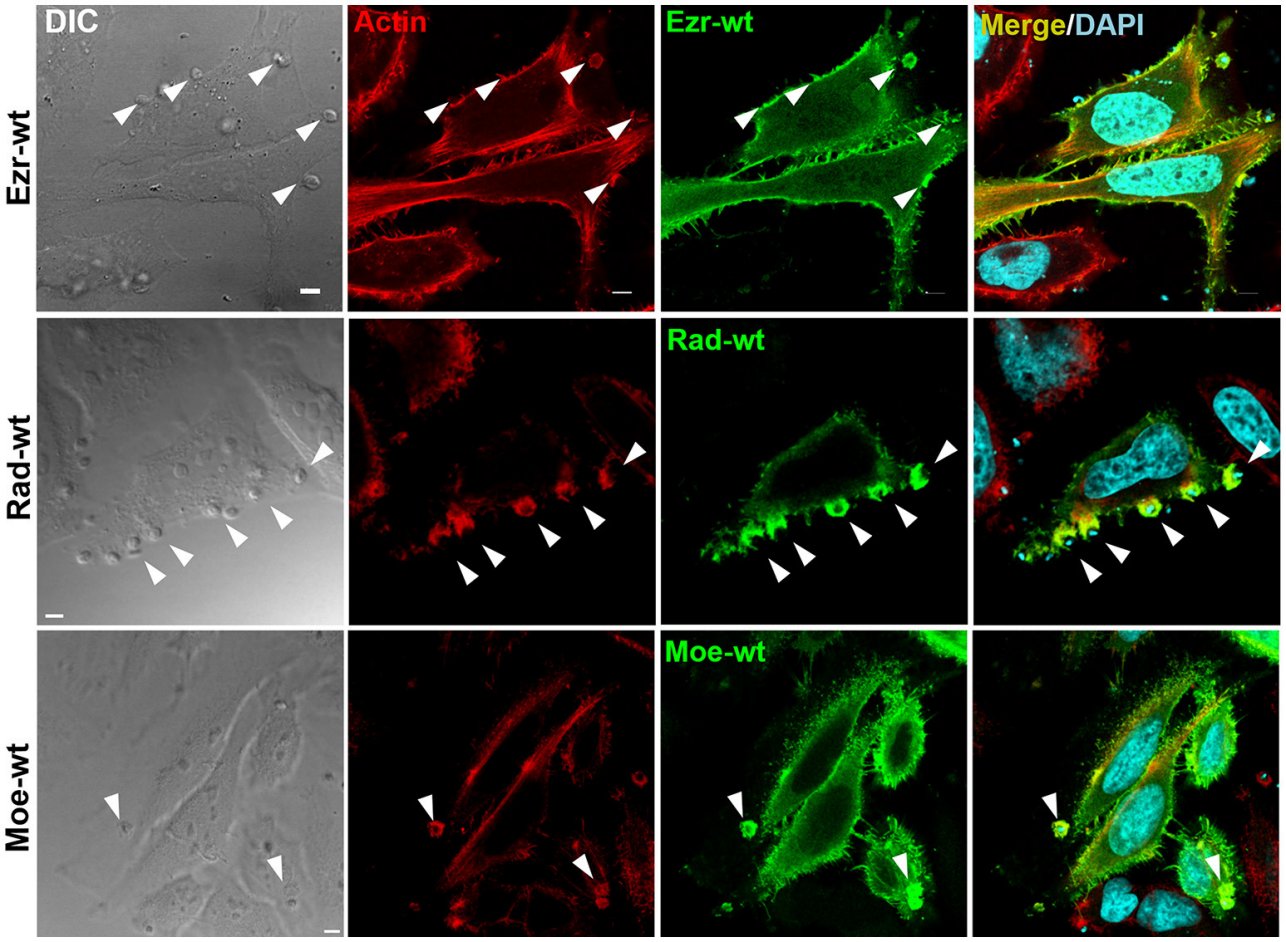
Ezrin, radixin, and moesin wild type are recruited to EAs invasion site. HeLa cells transfected with plasmids containing wild type ezrin-GFP, radixin-HA or moesin-GFP were incubated with EA for 1 h, fixed and incubated with phalloidin-TRITC to stain filamentous actin (red). DAPI was used to stain nuclei and kinetoplasts (cyan). For radixin visualization we employed anti-HA immunofluorescence protocol, since radixin constructs are HA-tagged. Arrowheads indicate EA invasion sites. Single plane images were acquired using confocal microscopy. Bar: 5 μm.

Considering the importance of protein recruitment to EAs invasion sites (Procópio et al., 1999; Bonfim-Melo et al., 2015), HeLa cells were transfected with plasmid containing wild type GFP-tagged ezrin and moesin or HA-tagged radixin (detected by anti-HA antibodies). Using confocal microscopy we observed the recruitment of ezrin, radixin and moesin colocalizing with F-actin to EAs invasion sites (Figure 2).

**Figure 3:**
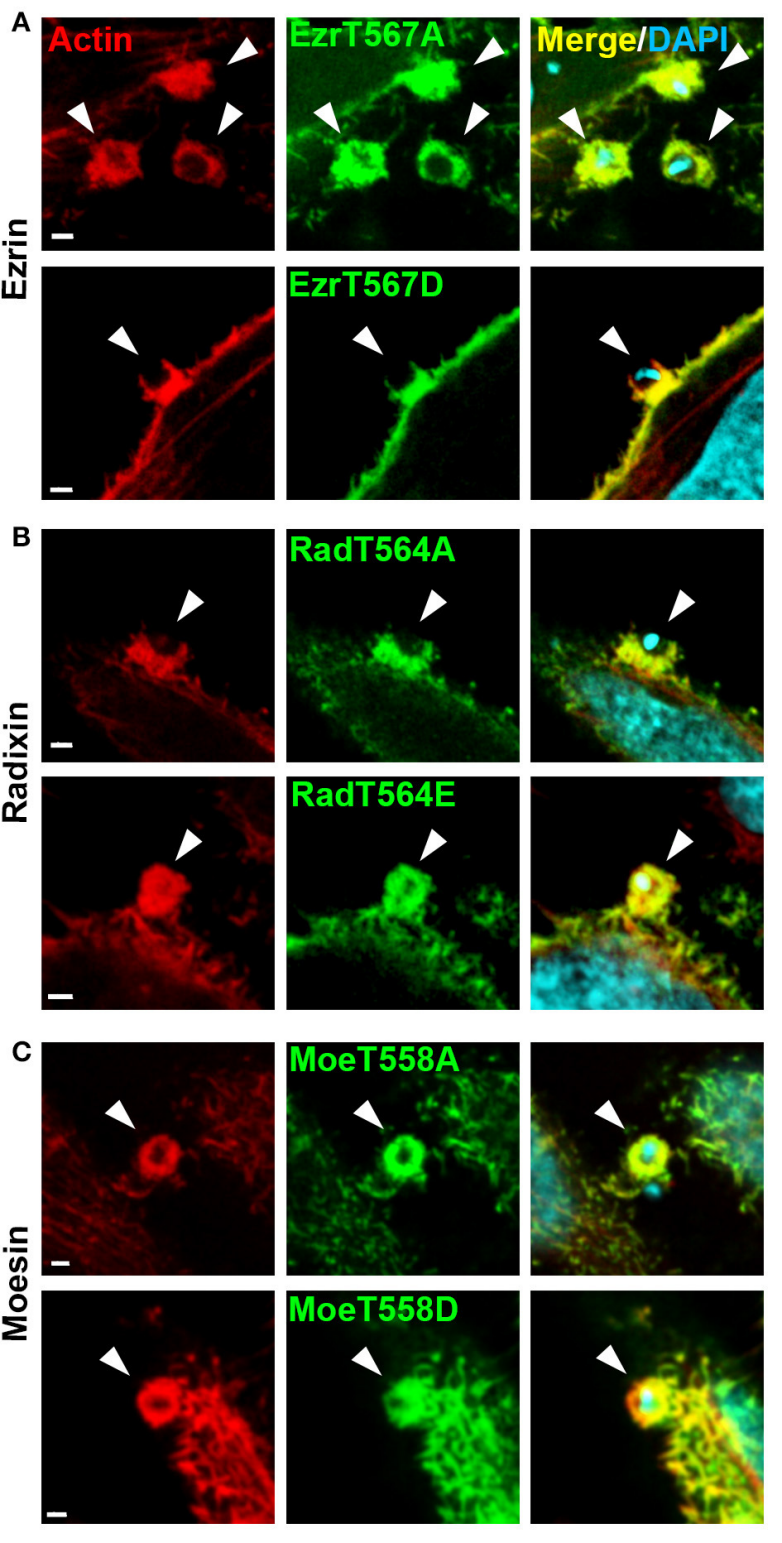
ERM proteins with inactive or active C-terminal threonine residue are recruited to EA invasion sites. **(A)** HeLa cells were transfected with ezrin-T567A (alanine replaced) or T567D (aspartic acid replaced), and incubated with EAs for 1 h. Next, cells were fixed and incubated with phalloidin-TRITC (red) to stain filamentous actin and DAPI to stain nuclei and kinetoplasts (cyan). **(B,C)** Similar experiments were performed using radixin and moesin with inactive or inactive C-terminal residue (T564A or T564E (glutamic acid replaced) to radixin and T558A or T558D to moesin). To visualize radixin we used anti-HA immunofluorescence protocol. Arrowheads indicate parasite invasion sites. Images were acquired using single plane confocal microscopy. Bar: 2 μm.

Phosphorylation at a C-terminal position is one of the best-studied mechanisms of ERM activation, crucial for their varied cellular activities (Ren et al., 2009; Cernuda-Morollon et al., 2010; Epting et al., 2015). ERMs with C-terminal threonine substitution by alanine (alanine replaced) are not activated by this mechanism and, without additional stimuli, remain in closed conformation while replacement of the same residue by acid amino acids (acidic replaced) mimic the phosphorylation state and are constitutively in opened conformation. To investigate the role of C-terminal phosphorylation during EA invasion we examined the recruitment of ERM mutated in this residue. EAs induced ERM and F-actin recruitment in cells expressing dephospho mimetic constructs (alanine replaced) similar to cells expressing ERM native isoforms and acidic replaced ERMs were also recruited along with actin to EA invasion sites (Figure 3).

**Figure 4:**
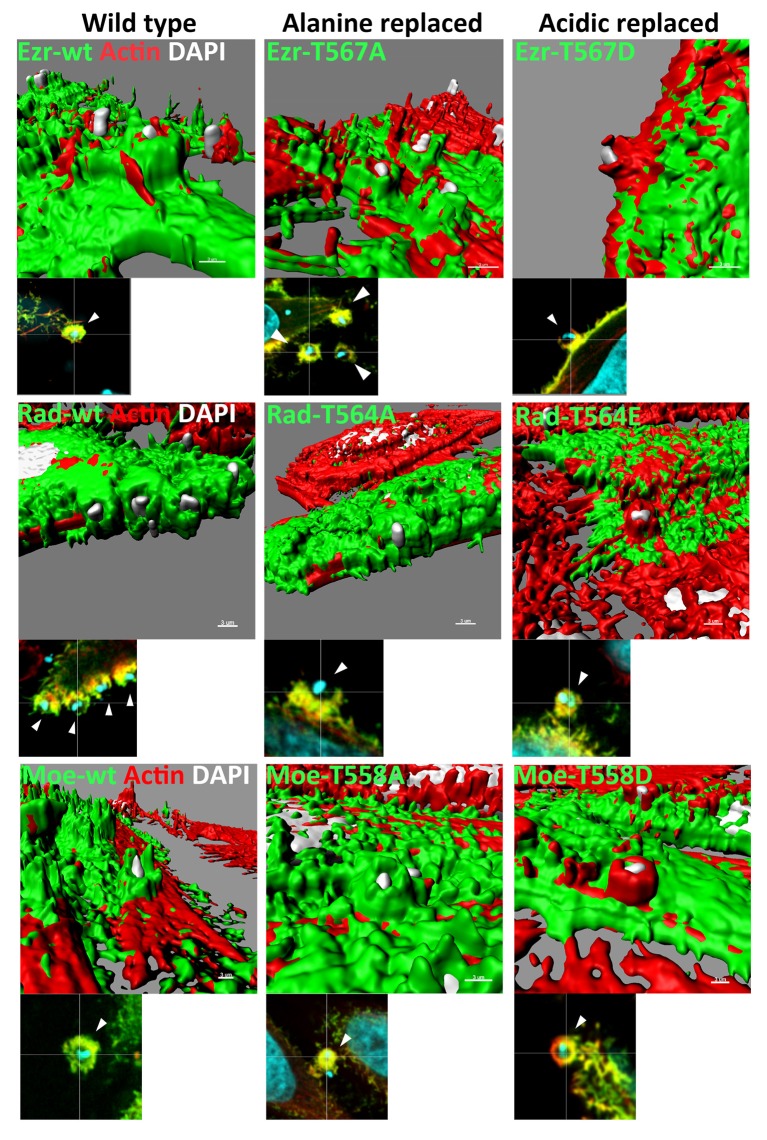
Acidic replaced ERM proteins are recruited to EA invasion site but localize more distant in relation to F-actin. Three-dimensional reconstruction based of fluorescence signal from ERM proteins with constitutively active C-terminal threonine residue-GFP or HA to radixin (green) stained with phalloidin-TRITC (red) and DAPI (nucleic acid marker, cyan). Different from wild type or inactive C-terminal phosphorylation residue (alanine replaced), ERM proteins with constitutively active C-terminal phosphorylation residue (acidic replaced) are more distant to EA invasion site in relation to F-actin. Three-dimensional reconstructions were performed using the surface tool from IMARIS software. Bar: 3 μm.

Although recruitment of constitutively active ERMs to EA invasion site could be detected by confocal analysis, detailed examination of three-dimensional surface reconstructions based on the fluorescence signal revealed that ERM proteins in this state are not recruited to EA invasion sites in the same way as wild type forms or constructs bearing inactive C-terminal phosphorylation residue. ERM-wt or inactive C-terminal phosphorylation residue constructs are closer to the parasite in relation to the surface of the HeLa cell. By contrast, in ERM constructions with constitutively active C-terminal phosphorylation residues, F-actin is closer to the parasite (Figure 4). These results indicated that permanent activation hinders ERM proper distribution and possibly their function.

### Invasion assays using cells over-expressing ERM proteins

**Figure 5:**
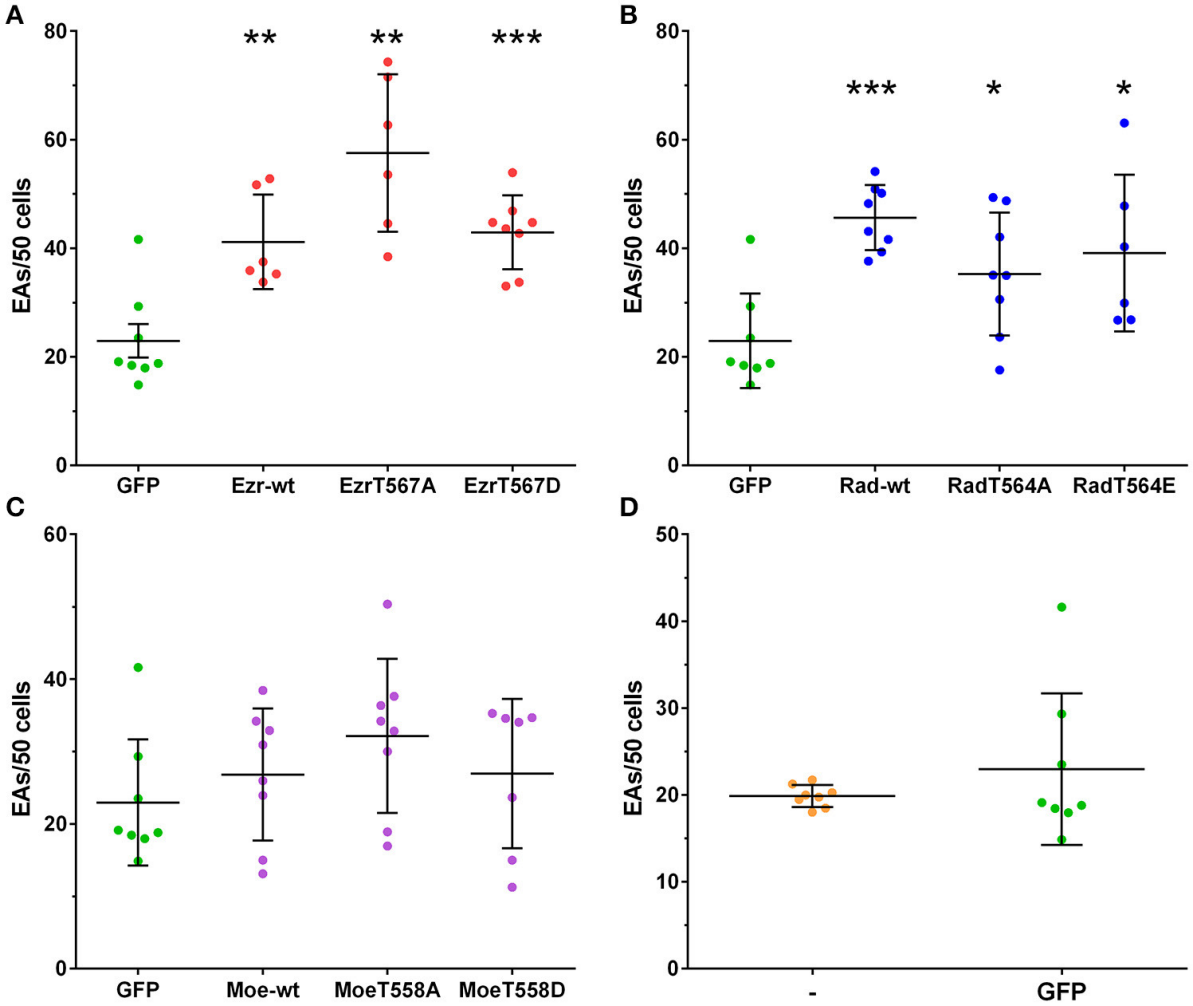
Overexpression of ezrin and radixin but not moesin increases EAs invasion rates. HeLa cells overexpressing ERM wild type and mutants were incubated with EAs for 2 h (MOI 10:1), fixed and mounted in coverslips for quantification under fluorescence microscopy. **(A–C)** EAs invasion rates in HeLa cells overexpressing ezrin, radixin or moesin wild type, inactive C-terminal residue (alanine residue) or constitutively active (acidic residue) compared to GFP transfected cells (GFP). For radixin (HA tagged), cells were submitted to immunofluorescence protocol. **(D)** Graph shows that GFP tag and transfection does not interfere in EA invasion rates compared to non-transfected cells (-). The experiment was performed in duplicate (two coverslips per group in each experiment) and intracellular parasites were counted in 50 cells/coverslip. This result is the mean and standard deviation of four independent experiments ±SD. ^*^P < 0.05, ^**^P < 0.005, and ^***^P < 0.0001.

Native and mutated ERMs recruitment assays lead us to question whether their expression would modulate EA internalization. Overexpression of native ezrin isoform increased EA internalization by HeLa cells when compared to GFP control group. For cells overexpressing alanine or acidic replaced ezrin constructions (T567A or T567D), we observed EA internalization similar to the ezrin-wt group (Figure 5A). Similar to ezrin groups, the three radixin constructions (native, T564A and T564E) increased EA internalization in HeLa cells at similar levels (Figure 5B). By contrast, overexpression of moesin constructions (wt, T558A and T558D) did not affect EA internalization (Figure 5C). No significant differences in EA invasion rates were observed between GFP tag transfected cells and non-transfected control, indicating that transfection procedure does not interfere with this process (Figure 5D). These results showed that overexpression of ezrin and radixin but not moesin increases EA internalization independently from the phosphorylation state of the C-terminal region.

**Figure 6:**
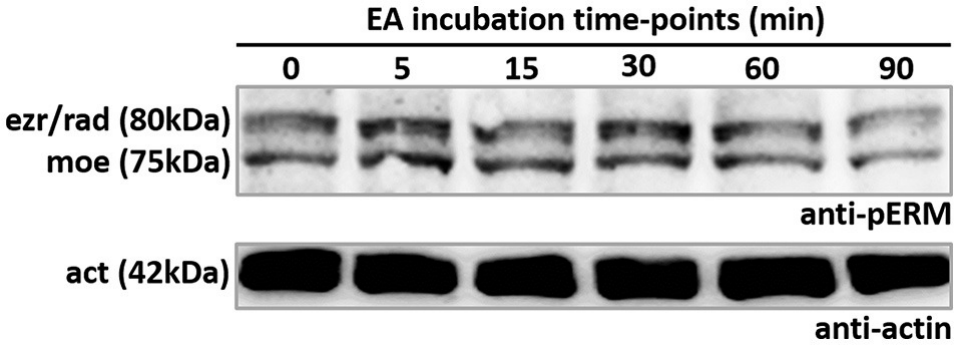
EA incubation does not alter phosphorylation of C-terminal threonine residue of ERM proteins. HeLa cells incubated with EAs from 0 to 90 min were lysed and submitted to SDS-PAGE and western blotting using anti-pERM antibodie. Results revealed that ERM phosphorylation is not affected in cells incubated with EAs. Representative image of three independent experiments.

In order to evaluate the role of EA interaction in ERM proteins activation, we incubated HeLa cells with EAs for different time points (5–90 min). We observed no increase in phosphorylation of C-terminal threonine residue of ERM proteins during EA incubation for the indicated time points (Figure 6). This result showed that EAs incubation does not trigger ERM phosphorylation, indicating that an alternative activation mechanism may be involved during EA host cell invasion.

### Actin dynamics during EA interaction

**Figure 7:**
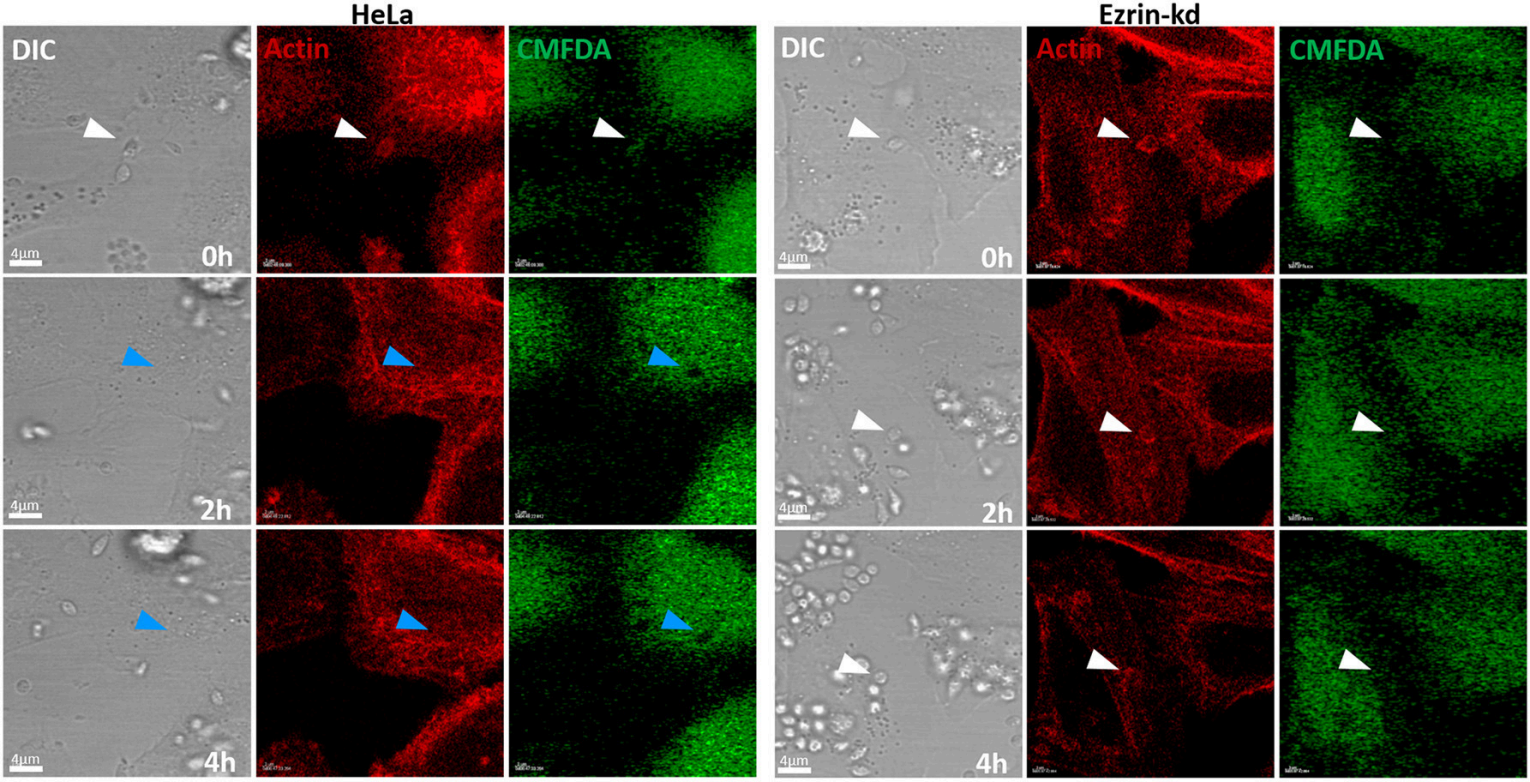
Ezrin-kd display apparent delayed EA internalization after actin recruitment to invasion site. Using LifeAct-RFP® to stain F-actin of live cells (red) and CMFDA (green; used to monitor parasite entry by the formation of dark halos in the cytoplasm), we submitted ezrin-kd cells to confocal time-lapse experiments. The images revealed that 2 h after EA interaction begins parasites were already inside cells (White arrows indicate parasite position while attached to host cell membrane, that turn into blue ones when parasite is internalized) in control groups (HeLa), while in ezrin-kd group, even after 4 h of interaction, parasites were not internalized and still recruit actin. Bar: 4 μm.

Given that ezrin and radixin depletion inhibits EA invasion in HeLa cells, we investigated whether depletion of ERM proteins would affect actin dynamics during EA internalization. To address this question we performed time-lapse experiments using cells depleted for ezrin, radixin or moesin transfected with LifeAct-RFP® that stains F-actin in live cells. The initial experiments indicated that ezrin depleted cells delay parasite invasion after actin mobilization to the invasion site, compared to non-transduced HeLa cells (Figure 7).

**Figure 8:**
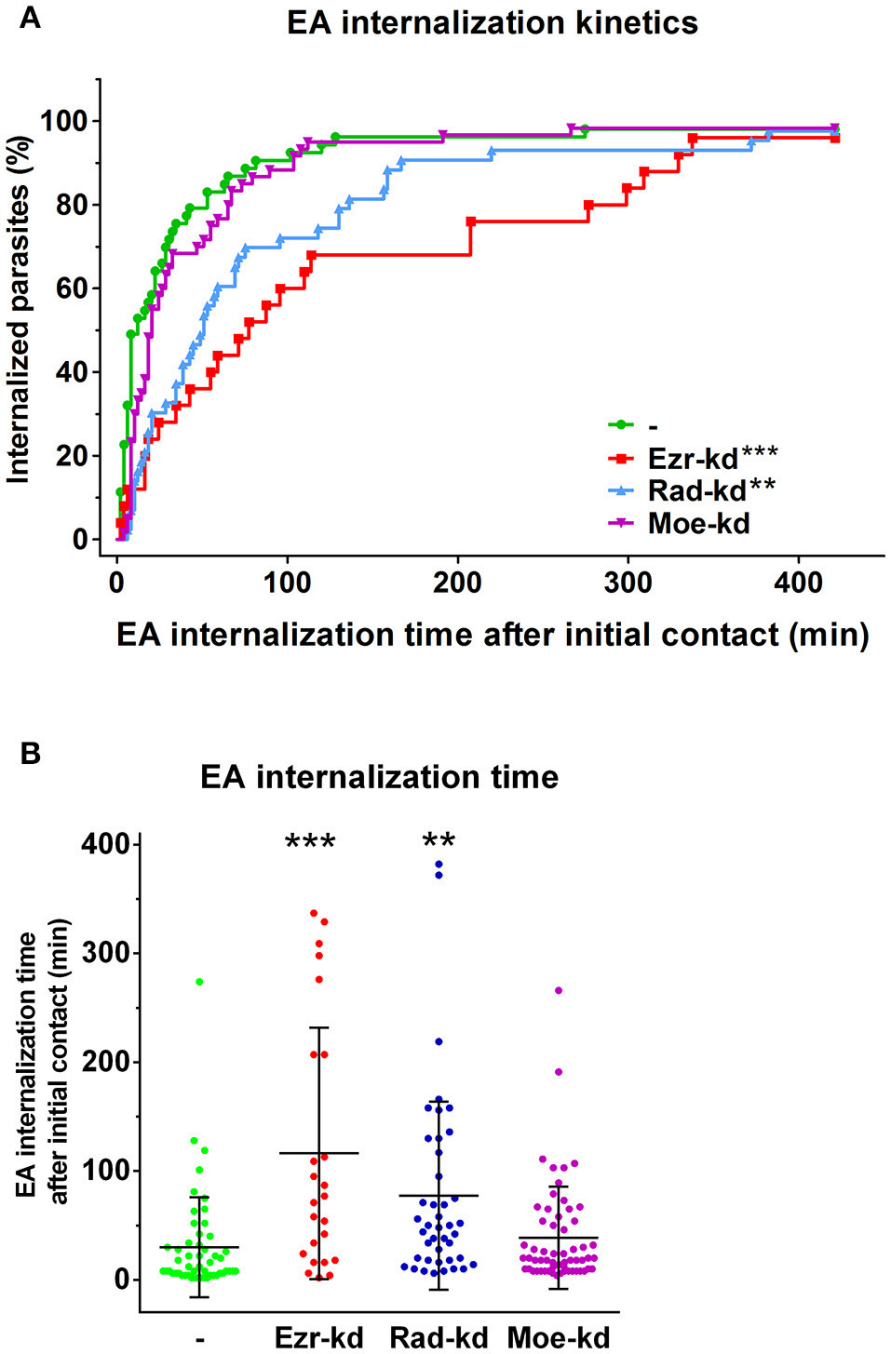
HeLa cells depleted for ezrin or radixin present a delay between actin recruitment and parasite invasion. **(A)** Percentage of internalized parasites over time. Ezr-kd and Rad-kd groups show delayed parasite internalization after attachment, related to control group (-). No differences were found in Moe-kd group. **(B)** Dispersion graph with the same data from **(A)** with mean time of internalization. Quantification of actin-recruiting parasites and internalization moment were carried out after LifeAct-RFP® transfection and cell labeling with CMFDA (used to monitor parasite entry by the formation of dark halos in the cytoplasm). Statistical data were generated by the comparison between control and shRNAi groups of the internalization time of individual parasites (in minutes) since the initial contact until internalization, by the same cell. These results are the mean and standard deviation of two independent experiments ±SD. ^**^P < 0.005 and ^***^P < 0.0001.

Since our observations showed that ezrin-kd cells delayed EA internalization after actin recruitment we performed similar experiments with ezrin, radixin and moesin depleted cells and quantified the time points of parasite attachment to the cell membrane, beginning of actin recruitment, and internalization time of individual parasites. These experiments revealed that ezrin and radixin-kd cells present delayed parasite internalization after actin recruitment to the interaction site. Figure 8A demonstrates the percentage of parasites internalized over time (minutes) and Figure 8B displays a dispersion graph with the same data from Figure 8A with mean time and standard deviation of EA internalization. This phenomenon was not observed in moesin-kd group (Figure 8, Videos 1–4, Supplementary Figure 2).

Live cell time-lapse experiments also enabled us to verify whether actin recruitment after initial contact was affected in our ERM-kd cells. We observed that only moesin-kd cells present delayed actin recruitment after initial parasite contact (Supplementary Figure 1). These results showed that in ezrin and radixin-kd groups actin dynamics during parasite interaction is functional, but with alterations that prevent normal parasite internalization. Differently, moesin-kd group presented a delayed actin recruitment but no delay in overall parasite internalization kinetics.

## Discussion

Cell invasion by *T. cruzi* is driven by complex and orchestrated signaling events that involve surface and secreted molecules from the protozoan which in turn trigger host cell responses leading to parasite invasion (reviewed in Caradonna and Burleigh, 2011; Ferreira et al., 2012; Maeda et al., 2012). Regarding host cell invasion by extracellular amastigote (EA) forms, the participation of F-actin dynamics driving parasite invasion is well established (Mortara, 1991; Procópio et al., 1998, 1999; Bonfim-Melo et al., 2015). Furthermore, not only F-actin and its regulating proteins are responsible for EA internalization but also host membrane components are required (Procópio et al., 1999). Linking these structures stand ERM proteins, known as interaction mediators of the actin cytoskeleton and plasma membrane (reviewed in Louvet-Vallee, 2000). To investigate the role of ERM proteins in EA invasion we used HeLa cells stably depleted for ERM proteins or overexpressing mutant constructions and evaluated by microscopy methods. Herein we demonstrated for the first time that despite structural similarities among ERM proteins, they may play distinct roles during *T. cruzi* infection.

EA invasion assays showed that depletion of ezrin or radixin, but not moesin, reduces EA internalization by HeLa cells. Further, corroborating these observations, in cells overexpressing GFP-tagged ezrin and radixin, but not moesin, an increased parasite uptake was observed. These results showed the importance of linking the actin cytoskeleton, the major element in EA uptake, to the plasma membrane, site of EA-host cell interaction. It seems that despite structural and functional similarities among ERM proteins each of them plays distinct roles during EA host cell invasion. Functional differences among ERM proteins were reported in KO mice for ezrin, radixin or moesin in which compensatory mechanisms among them may occur (Doi et al., 1999; Kikuchi et al., 2002; Kobold et al., 2002; Kitajiri et al., 2004; Saotome et al., 2004; Okayama et al., 2008; Hirata et al., 2012). For example, ezrin replaces the lack of radixin in stereocilia of auditive vestibule cells preventing spatial imbalance in radixin KO mice (Kitajiri et al., 2004). Our experiments indicate that moesin does not participate in EA invasion or a compensatory mechanism by ezrin and/or radixin may develop in moesin absence. Although compensatory mechanisms may occur in HeLa cells depleted for moesin, this phenomenon does not seem to occur in ezrin or radixin depleted cells that presented reduced EA invasion when compared to control groups suggesting that ezrin and radixin may play an exclusive role during this event.

Our time-lapse confocal microscopy experiments showed that F-actin polymerization and mobilization occurred with similar dynamics in cells depleted for ERM proteins compared to non-transduced cells. This corroborates with the fact that ERM proteins mediate filamentous actin-plasma membrane interaction but it is not clear whether ERM proteins alone are directly involved in actin polymerization (Crepaldi et al., 1997; Mackay et al., 1997; Naba et al., 2008; Arpin et al., 2011). On the other hand, these experiments showed that in ERM depleted cells EA internalization is delayed. Similar results were found in experiments with lysosome-phagosome fusion. The work of Marion et al. (2011) reports that ezrin is critical for F-actin assembly during lysosomal fusion with phagosomes. Interestingly, this work shows that ezrin inactivation does not affect lysosome dynamics toward phagosomes (also an F-actin dependent process; Taunton et al., 2000), while fusion with phagosomes is impaired. These findings are in line with our live cell analyses that revealed no alterations in F-actin dynamics at EA adhesion sites in ezrin and radixin depleted cells, but in F-actin assembly and/or stabilization required for internalization. Altogether, these results suggest that ERM proteins do not participate in initial actin recruitment but in its reorganization required for EA internalization.

Recruitment and colocalization of ezrin with actin by *Neisseria meningitidis* marks the activation of proper signaling pathways during host cell invasion by these bacteria (Lambotin et al., 2005). In our work, recruitment assays revealed that the three ERM proteins are recruited to EA adhesion sites and colocalize with F-actin indicating that these proteins may interact during EA invasion process. This result indicates that moesin has sufficient structural requirements for its recruitment. Despite no significant differences were observed in EA invasion in moesin depleted cells, cooperative activity of moesin with ezrin or radixin at these sites cannot be ruled out (Paglini et al., 1998).

Phosphorylation of C-terminal threonine residue of ERM proteins causes conformational changes resulting in their activation (Nakamura et al., 1995). In the present work, we observed that this mechanism might not be related to EA internalization since cells expressing mutants at this phosphorylation site were similarly invaded by EAs when compared to wild type constructs. Assays with these constructs showed recruitment of dephosphomimetic (threonine to alanine substitution) isoform suggesting that activation and recruitment to EA invasion sites occurred independent of C-terminal phosphorylation. Similar behavior was described in osteosarcoma cells in which binding to F-actin by C-terminal inactive ezrin occurs similarly to the wild type ezrin (Ren et al., 2012). On the other hand, phosphomimetic (threonine to acidic amino acid substitution) isoforms were spatially farther to EA invasion sites when compared to actin-rich cups possibly because constitutively opened state of these isoforms led them to bind to the plasma membrane in other sites (Supplementary Figure 3). Corroborating this data, constitutive activation of C-terminal phosphorylation residue induces ERM proteins to be addressed strictly to the plasma membrane that impairs correct protein responses to normal stimuli (Cernuda-Morollon et al., 2010; Ren et al., 2012). Osteosarcoma cells transfected with C-terminal constitutively active ezrin are not able to form primary tumors and its migration to other tissues (metastasis) is inhibited (Ren et al., 2012). Another work from the same group demonstrated that in osteosarcoma cells activation and inactivation of ezrin are essential for tumor growth and migration (Ren et al., 2009). It has also been described that regulation between active and inactive ezrin is required for normal growing of *Xenopus laevis* and *Danio rerio* ciliated cells (Epting et al., 2015). Additionally, overexpression of ezrin with constitutively active or inactive C-terminal residue compromises the chemotactic activity of B lymphocytes and that the immunological synapsis of T lymphocytes is dependent on regulated phosphorylation and dephosphorylation of ERM proteins (Faure et al., 2004; Cernuda-Morollon et al., 2010; Parameswaran et al., 2011). Moreover, there are reports showing that C-terminal phosphorylation is not crucial for ERM activation and function during infection by the intracellular pathogen protozoan *Theileria annulata* (Baumgartner, 2011). Finally, in our work, EA incubation with HeLa cells revealed no alteration in phosphorylation at this residue (Figure 6) corroborating that C-terminal threonine phosphorylation is not essential for ERM protein activity during EA invasion. A possible mechanism that leads to ERM proteins activation during EA invasion could be the binding of PIP_2_ to ERM proteins. PIP_2_ binding induces conformational changes from closed to opened state and consequent activation comparable to that following C-terminal threonine phosphorylation (Hao et al., 2009; Bosk et al., 2011). We speculate that this activation mechanism is likely to occur in ERM activation during EA internalization due coordinated phosphoinositide recruitment during phagocytosis-like process induced by these forms (Fernandes et al., 2013). Finally, ERM proteins with constitutively active C-terminal residue can be unbound from the membrane (not to the same extent as the wild type ERM) by PLC activity leading to PIP_2_ hydrolysis, a mechanism that provides activity and localization dynamics to these proteins (Hao et al., 2009). However, we cannot rule out the possibility of a yet unknown mechanism that lead to the activation of ERM proteins during EA invasion process.

Thus, here we showed that ezrin and radixin play similar a role in EA host cell invasion mediating actin binding to plasma membrane and participate in actin dynamics necessary for EA internalization. Compensatory mechanisms among ezrin, radixin and moesin may occur and their activity during EA invasion is independent of C-terminal threonine phosphorylation.

## Author contributions

Conception of the study: RM, EF, Designed the experiments: EF, AB-M, and RM. Performed the experiments: EF, AB-M, and EC. Interpretation of the results: EF, AB-M, and RM. Wrote the manuscript: EF, AB-M, and RM.

### Conflict of interest statement

The authors declare that the research was conducted in the absence of any commercial or financial relationships that could be construed as a potential conflict of interest.
