# Supplementary material for: ERM Proteins Play Distinct Roles in Cell Invasion by Extracellular Amastigotes of Trypanosoma cruzi
Source: Front Microbiol. 2017 Nov 21;8:2230. doi: 10.3389/fmicb.2017.02230 (PMC5702390; doi:10.3389/fmicb.2017.02230)
Supplement: Supplementary file 5 [file DataSheet1.PDF]

Supplementary Material

ERM proteins play distinct roles in cell invasion by extracellular amastigotes of *Trypanosoma cruzi*

Éden Ramalho Ferreira[1#], Alexis Bonfim-Melo[1#], Esteban Mauricio Cordero[1,2], Renato Arruda Mortara[1]

[1] Departamento de Microbiologia, Imunologia e Parasitologia, Escola Paulista de Medicina, Universidade Federal de São Paulo

[2] Present address: Centro de Genómica y Bioinformática, Facultad de Ciencias, Universidad Mayor, Santiago, Chile.

[#] **Joint first Authors**

*** Correspondence:**
R. A. Mortara; ramortara@unifesp.br

Supplementary Figures

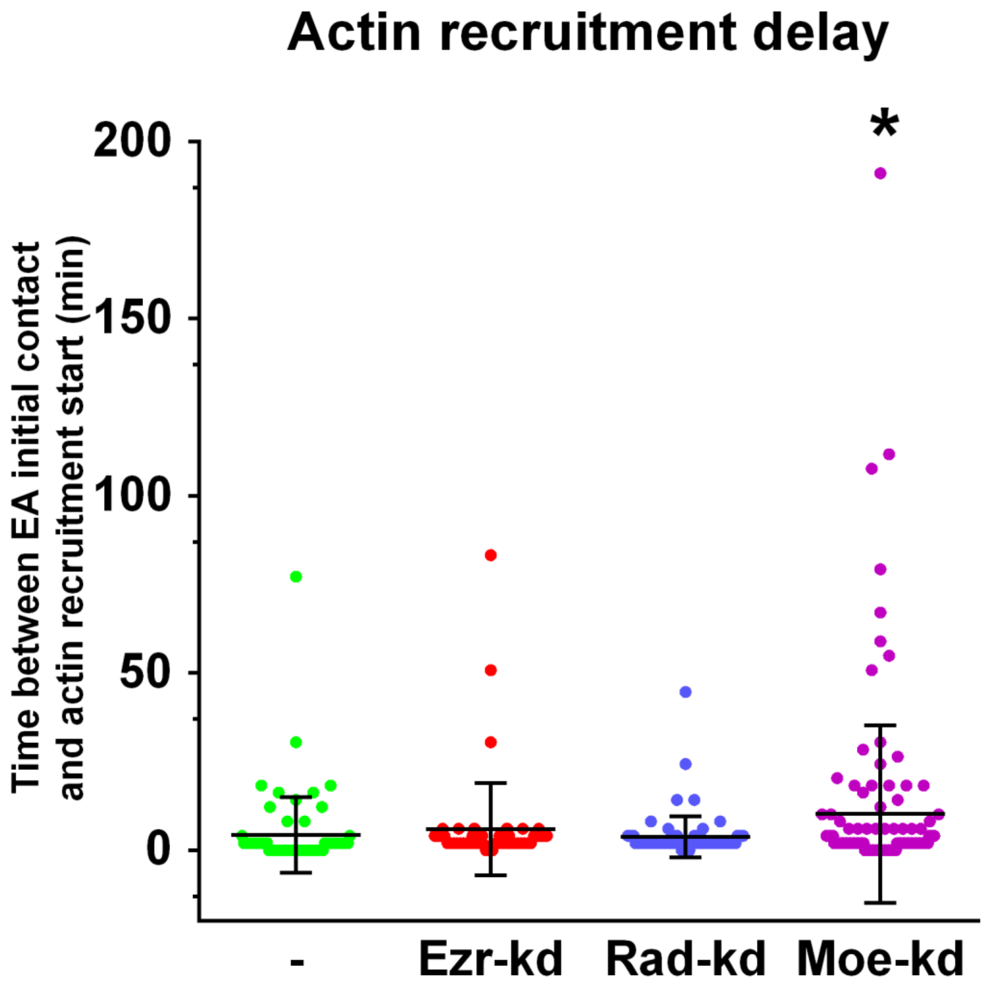

Supplementary Figure 1: Moesin depleted cells present delayed actin recruitment after parasite attachment. Live cell imaging experiments permitted the quantification of time-points from parasite attachment to actin recruitment start. Delay in actin recruitment to EA attachment site was observed only in Moe-kd group. This result is the mean of two independent experiments ± standard deviation (SD). * P <0.05. Statistical analysis was performed by Student T test method.

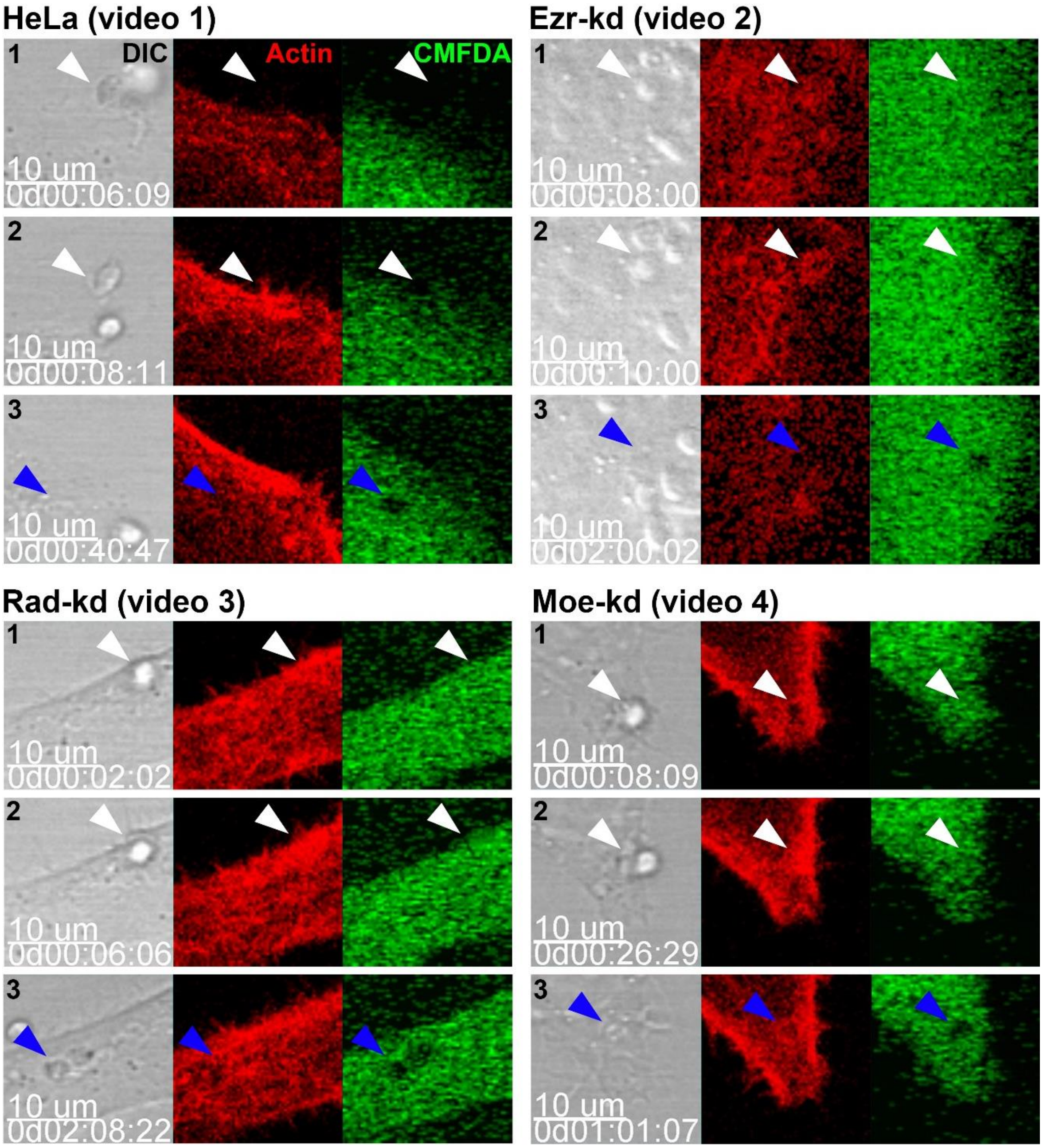

Supplementary Figure 2: Still images (from video1-4) of parasite initial contact (1), actin recruitment (2) and internalization (3).

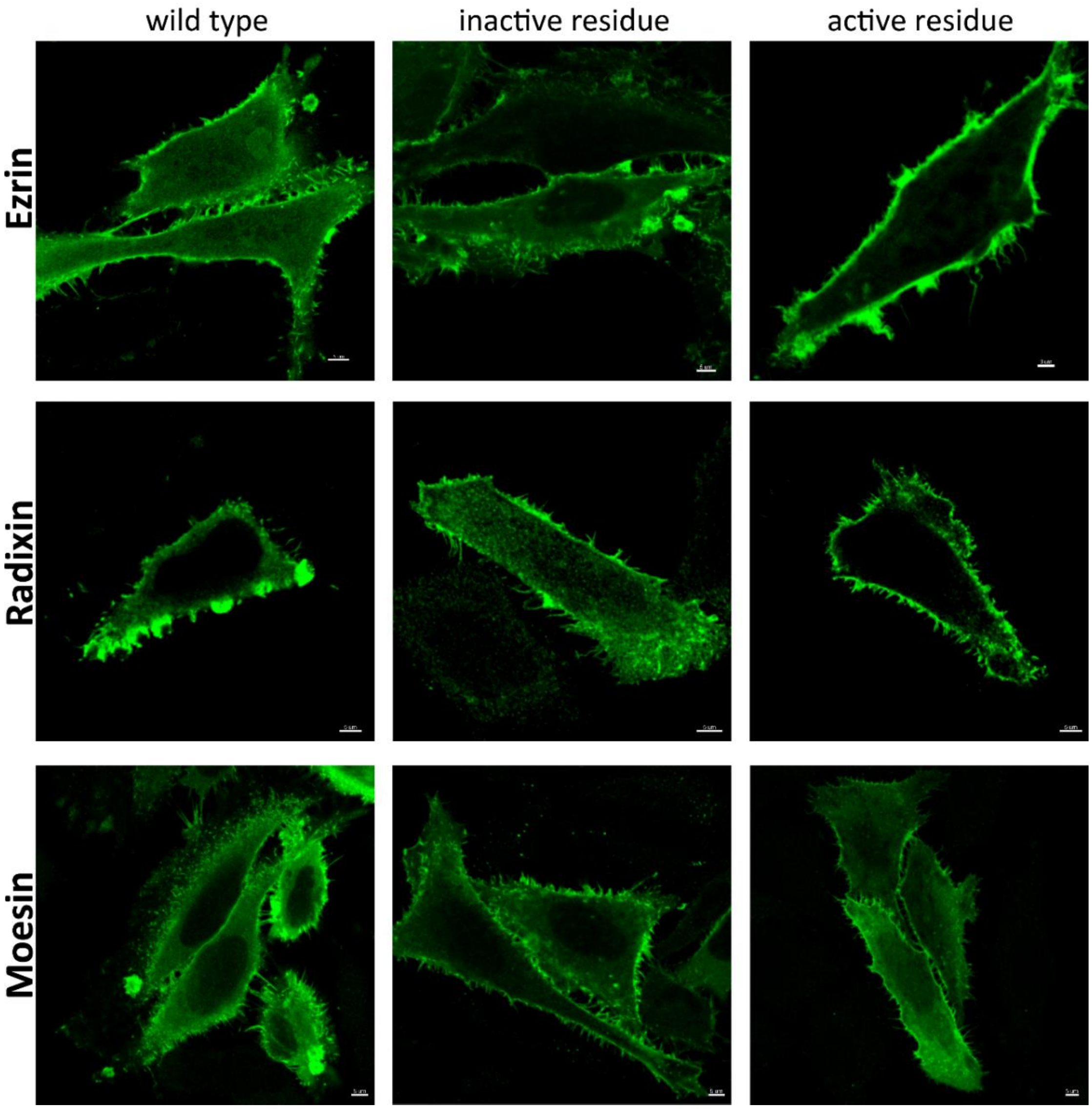

Supplementary Figure 3: Ezrin and radixin with constitutively active C-terminal residue localize mostly at plasma membrane. Analysis of confocal images revealed that besides differences in EA invasion site recruitment, ezrin and radixin with constitutive active residue localize to the plasma membrane, different from wild type or proteins with C-terminal inactive residue that are dispersed in the cytoplasm and plasma membrane. Differences in protein localization were not observed with moesin constructions. Bar: 5µm.

Videos

VIDEO 1-4: Representative interactions of EA internalization in non-transduced HeLa cells (video 1): parasite internalization occurs at 30 min, Ezr-kd (video 2): parasite internalization occurs at 120 min, Rad-kd (video 3): parasite internalization occurs at 90 min, Moe-kd (video 4): parasite internalization occurs at 50 minutes. Using LifeAct-RFP® to stain F-actin of live cells (red) and CMFDA (green; used to highlight parasite entry by the formation of dark halos in the cytoplasm), we submitted non-transduced HeLa and ERM depleted cells to confocal time-lapse experiments. White arrows indicate parasite position while attached to host cell membrane, that turn into blue ones when parasite is internalized. Bar: 10µm. Videos were prepared using IMARIS for

record, ImageJ and VirtualDUB for stack assembly and Sony Vegas for arrow insertion and final rendering. To assist video examination supplementary figure 2 displays still images from videos showing initial contact (1), actin recruitment (2) and internalization (3) time-points.
